# Sulodexide attenuates endoplasmic reticulum stress induced by myocardial ischaemia/reperfusion by activating the PI3K/Akt pathway

**DOI:** 10.1111/jcmm.14367

**Published:** 2019-05-23

**Authors:** Danping Shen, Ruiyao Chen, Lijing Zhang, Zhiheng Rao, Yongxue Ruan, Lei Li, Maoping Chu, Yuanhai Zhang

**Affiliations:** ^1^ Children's Heart Center Institute of Cardiovascular Development and Translational Medicine, The Second Affiliated Hospital & Yuying Children's Hospital of Wenzhou Medical University Wenzhou Zhejiang China; ^2^ The Children's Hospital Zhejiang University School of Medicine Hangzhou Zhejiang China

**Keywords:** endoplasmic reticulum stress, myocardial ischaemia / reperfusion, PI3K/Akt, sulodexide

## Abstract

Acute myocardial ischaemia/reperfusion (MI/R) injury causes severe arrhythmias with a high rate of lethality. Extensive research focus on endoplasmic reticulum (ER) stress and its dysfunction which leads to cardiac injury in MI/R Our study evaluated the effects of sulodexide (SDX) on MI/R by establishing MI/R mice models and in vitro oxidative stress models in H9C2 cells. We found that SDX decreases cardiac injury during ischaemia reperfusion and decreased myocardial apoptosis and infarct area, which was paralleled by increased superoxide dismutase and reduced malondialdehyde in mice plasm, increased Bcl‐2 expression, decreased BAX expression in a mouse model of MI/R. In vitro, SDX exerted a protective effect by the suppression of the ER stress which induced by tert‐butyl hydroperoxide (TBHP) treatment. Both of the in vivo and in vitro effects were involved in the phosphatidylinositol 3‐kinase (PI3K)/Akt signalling pathway. Inhibition of PI3K/Akt pathway by specific inhibitor, LY294002, partially reduced the protective effect of SDX. In short, our results suggested that the cardioprotective role of SDX was related to the suppression of ER stress in mice MI/R models and TBHP‐induced H9C2 cell injury which was through the PI3K/Akt signalling pathway.

## 1 INTRODUCTION

Myocardial ischaemia/reperfusion (MI/R) injury occurs when an aortic cross‐clamp is removed or a balloon is deflated, during cardiac surgery.^1^ It results in endothelial dysfunction, severe arrhythmias, apoptosis or necrosis, myocardial stunning and a high mortality rate.^2^, ^3^ Furthermore, there is no effective therapy for MI/R^4^ The underlying mechanisms of MI/R injury include calcium overload, free radical damage, microvascular damage and leucocyte activation,^5^, ^6^ which lead to endoplasmic reticulum (ER) and mitochondrial injury. Myocardial ischaemia/reperfusion injury induces the production of free radicals, leading to oxidative stress, denaturation, degradation and crosslinking of proteins and polysaccharides and ultimately apoptosis of cardiomyocytes.^7^ Therefore, inhibition of apoptosis induced by oxidative stress may be beneficial for the treatment of MI/R injury.

Glycosaminoglycans (GAGs) ameliorate injury to and maintain the contractile function of, ischaemic myocardium.^8^, ^9^ Sulodexide (SDX) is a GAG purified from porcine mucosa that comprises 80% low‐molecular weight heparin and 20% dermatan sulphate and exerts anti‐inflammatory and lipid‐lowering effects.^10^, ^11^ Sulodexide is used for the treatment of chronic venous^12^ and arterial diseases.^13^ Although SDX prevents arterial thrombosis,^14^ inhibits the proliferation of smooth muscle cells,^15^ and reduces the pro‐inflammatory effect of peripheral arterial disease in human arterial endothelial cells in patients with atherosclerosis,^16^ its effects on MI/R are unclear. Following oral administration, SDX is degraded into N‐acetyl‐D‐glucosamine in the digestive tract, resulting in a significant increase in the levels of GAG precursors, up to 18% of which are absorbed.^17^, ^18^

Intracellular signal transduction pathways play an important role in MI/R. Phosphatidylinositol 3‐kinase (PI3K) and Akt are signal transduction proteins critical for many aspects of cell survival, growth and apoptosis. They are reportedly involved in the pathological processes of diverse diseases.^19^ In the myocardium, PI3K/Akt indirectly regulates contraction of cardiac muscle, calcium channels^20^ and myocardial ischaemic reperfusion.^21^ It is also implicated in the control of cell growth, proliferation,^22^ apoptosis^23^ and ER stress.^24^, ^25^ Lauver et al reported that SDX attenuates MI/R injury.^26^ However, the relationships of SDX to myocardial injury, ER stress‐induced apoptosis and the PI3K/Akt pathway are unknown.

We investigated the effects of SDX on myocardial ER stress and cardiomyocyte apoptosis in the heart of mice with MI/R and in H9C2 cells. In addition, the role of SDX in the PI3K/Akt signalling pathway was investigated using the PI3K/Akt inhibitor LY294002.

## 2 MATERIAL AND METHODS

### 2.1 Animals and materials

Male C57/B6 mice aged 8‐12 weeks were supplied by the Animal Center of the Wenzhou medical university. The animal use and care protocols were in line with the National Institutes of Health Guide of the Care and Use of Laboratory Animals and were approved by the Animal Care and Use Committee of Wenzhou medical University (Number: 2017‐010). Rat cardiomyocyte H9C2 cells were purchased from the American Type Culture Collection. DMEM and foetal bovine serum (FBS) were purchased from Invitrogen (Carlsbad, CA). Anti‐p‐PI3K (ab182651) was purchased from Abcam (Cambridge, MA). Anti‐PI3K (C73F8), anti‐AKT (C67E7), p‐AKT (Ser473), anti‐BAX (D3R2M), Bcl‐2 (D17C4), anti‐CHOP (D46F1), GRP‐78 (C50B12), ATF‐6 (D4Z8V), GAPDH (D16H11), β‐actin (13E5) antibodies and goat anti‐rabbit secondary antibody were purchased from Cell Signaling Technology, Inc (Danvers, MA). An enhanced chemiluminescence (ECL) kit was purchased from Bio‐Rad (Hercules, CA). Tert‐butyl hydroperoxide (TBHP) and PI3K/AKT inhibitor (LY294002) were purchased from Sigma‐Aldrich.

### 2.2 Cardiac I/R model in mice and treatment

The MI/R in mice was induced by transient myocardial ischaemia for 30 minutes followed by reperfusion for 2 hours. Specifically, mice were anaesthetized by inhaling isoflurane. The heart was completely exposed in the intercostal space by a lateral cut along the third or fourth rib. Then the left anterior descending coronary artery (LAD) was found and was ligatured. The heart grew grey quickly after using a 7‐0 silk suture at the distal 1/3 of the LAD.

The ligation was released after coronary artery occlusion for 30 minutes and reperfusion for 2 hours. The Sham group mice were also anaesthetized and the heart was exposed, a silk suture was passed under the LAD but not ligated. The MI/R mice were administered with 60 µg SDX/mouse through gastric administration at 30 minutes before ischaemia.^27^ The PI3K inhibitor LY294002 was administered into mouse through intramyocardial injection at 30 minutes before ischaemia and the dose is 0.2 mg/mouse.

### 2.3 Myocardial infarct size measurement

The myocardial infarct size was assessed by Evan's Blue and triphenyltetrazolium chloride (TTC) double staining method. At the end of reperfusion, the LAD was reoccluded and 0.2 mL of a 2% Evan's Blue dye was injected into the inferior vena cava. When the right side of the heart turned blue, the heart was quickly removed, rinsed in NS and frozen at −20°C. Five 1‐mm thick heart sections were made and incubated in 1% TTC for 20 minutes at 37°C. The viable tissue which was stained red by TTC was defined as area at risk (AAR). The non‐ischaemic myocardium was stained deep blue by Evans Blue. Infarct area (INF) appeared pale after staining. A percent of infarcted area over total AAR (% INF/AAR) was calculated.

### 2.4 Myocardial TUNEL staining

After 2 hours of reperfusion, the heart was removed. The heart tissues were then embedded in optimal cutting temperature compound and approximately 5‐µm thick sections were cut. Terminal deoxynucleotidyl transferase‐mediated dUTP nick end labelling (TUNEL) (Roche, Mannheim, Germany) staining was used to detect cardiomyocytes apoptosis according to the kit's manual. The section images were taken by a Nikon ECLIPSE Ti microscope (Olympus BX51, Japan).

### 2.5 Determination of superoxide dismutase activity and malondialdehyde levels

The superoxide dismutase (SOD) activity and malondialdehyde (MDA) levels were measured in mice plasm, which were conducted using commercial kit reagents (JianCheng Bioengineering Institute, Nanjing, China) according to the manufacturers' instructions.

### 2.6 Cell viability

H9C2 cells were cultured in high‐glucose DMEM with 10% (v/v) FBS without penicillin/streptomycin in a humidified atmosphere (5% CO_2_, 21% O_2_) at 37°C. H9C2 cells were seeded into 96‐well plates at a concentration of 5000 cells per well. The cells were pre‐treated with SDX at different concentration for 30 minutes before being exposure to TBHP for 12 hours. The PI3K inhibitor LY294002 was added to the cell at the concentration of 15 µmol/L for 30 minutes before adding TBHP. The number of viable cells was evaluated by CellTiter 96^®^ AQueous One Solution Cell Proliferation Assay (MTS). Briefly, 20% MTS dye solution was added to each well and incubated for 2.5 hours. The number of viable cells was measured by evaluating absorbance at 480 nm. The MTS assay was repeated three times for consistency.

### 2.7 Cell apoptosis assays

H9C2 cells (2 × 10^5^) were plated in 6‐well plates for 24 hours. After different treatments (note: LY294002 and SDX added at the same time), cells of each well were collected, washed twice with PBS and resuspended with 200 µL PBS. Then cells (100 µL) were incubated with 5 µL annexin V‐fluorescein isothiocyanate for 5 minutes, followed by 3 µL propidium iodide (PI) for 10 minutes in the dark at room temperature. The fluorescence intensities of annexin V/PI‐stained cells were analysed by a flow cytometry. The apoptosis ratio was quantified by BD FACS software.

### 2.8 Western blot

Total proteins in the heart tissue and H9C2 cells were extracted and denatured using protein extraction reagents. Fifty microgram of protein was then loaded in lanes of 10% gels, separated by electrophoresis and then transferred onto a polyvinylidene fluoride membrane. After blocking with 5% fat‐free milk, the membranes were incubated with the relevant primary antibodies overnight on a refrigerator shaker. The membranes were washed three times with TBS Tween and incubated with the relevant secondary antibodies at room temperature for 2 hours. The signals were visualized using ECL reagents. The amounts of the proteins were analysed using Image Pro Plus and normalized to their respective controls.

### 2.9 Statistical analysis

The data in this study are shown as the mean ± SEM Differences among groups were analysed using one‐away ANOVA accompanied with Turkey multiple‐comparisons test (IBM spss statistical 21). Two‐tailed Student's *t* test was used for comparison between two groups; *P* < 0.05 was considered significant.

## 3 RESULTS

### 3.1 SDX pre‐treatment decreased MI/R injury

**Figure 1:**
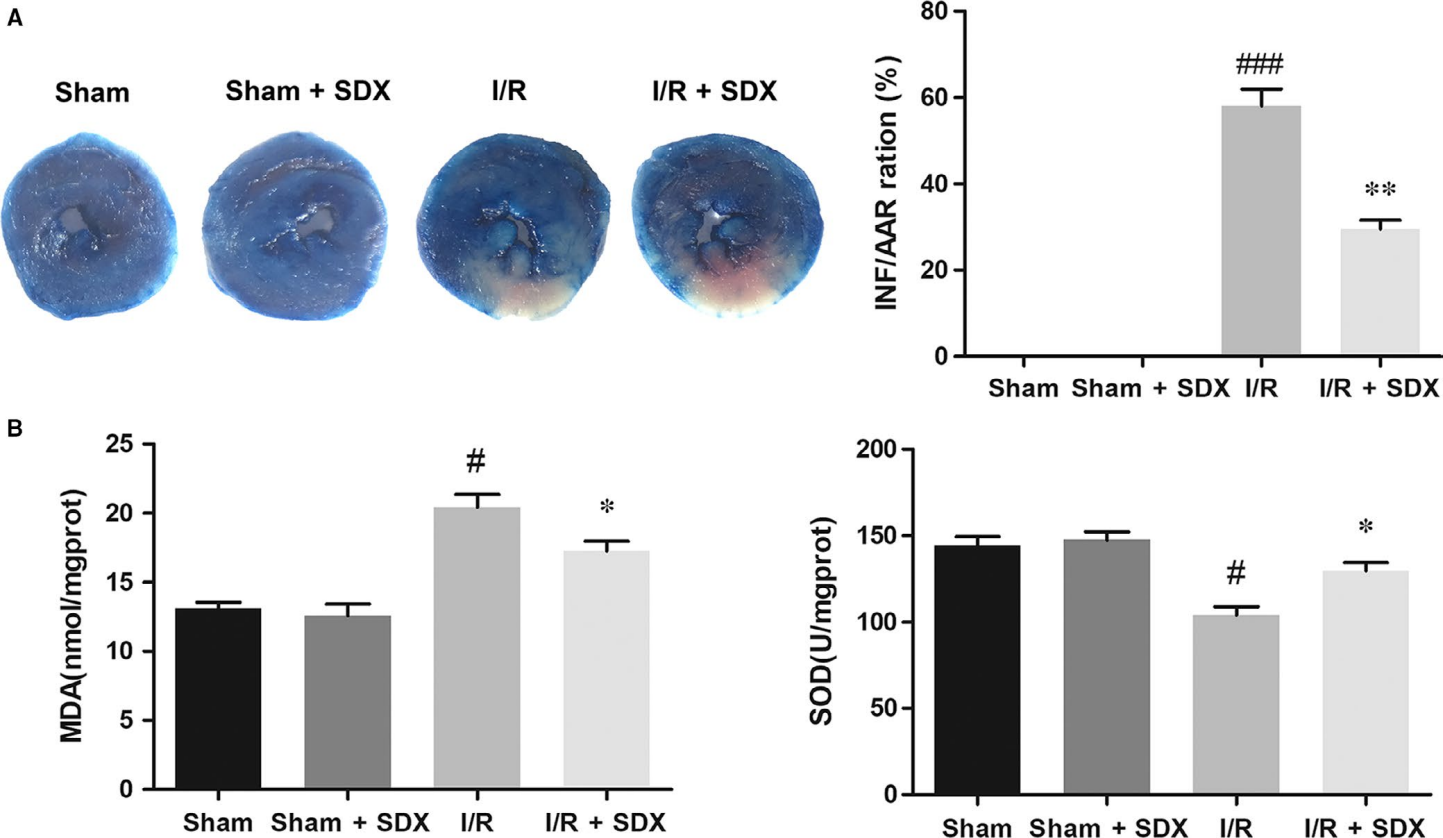
Sulodexide (SDX) pre‐treatment reduces myocardial ischaemia/reperfusion injury. A, Representative images and quantitative analysis of the infarct area (INF: white), area at risk (AAR: red and white) and normal area (blue). B, Superoxide dismutase activities and malondialdehyde leaves in mice serum. Data are shown as means ± SEM; ^#^ *P* < 0.05, ^###^ *P* < 0.001, I/R (pre‐treated with normal saline) control group vs Sham group; *P < 0.05, **P < 0.01, I/R + SDX (pre‐treated with SDX 60 µg) group vs I/R control group; n = 3 per group

To determine the role of SDX in cardiac protection, SDX was administered to mice 30 minutes before ischaemia. After 30 minutes of ischaemia and 2 hours of reperfusion, the size of myocardial infarcts was measured by TTC staining. Compared with the MI/R group, the infarct size was greatly reduced in the SDX pre‐treatment group (Figure 1A). Myocardial ischaemia/reperfusion injury affects ER integrity and promotes ER stress.^28^, ^29^ The level of MDA in plasma was markedly increased by MI/R and this effect was significantly reduced by SDX pre‐treatment. In contrast, the level of SOD was decreased by MI/R and this effect was reversed by SDX pre‐treatment (Figure 1B).

### 3.2 SDX pre‐treatment inhibited MI/R‐induced apoptosis

**Figure 2:**
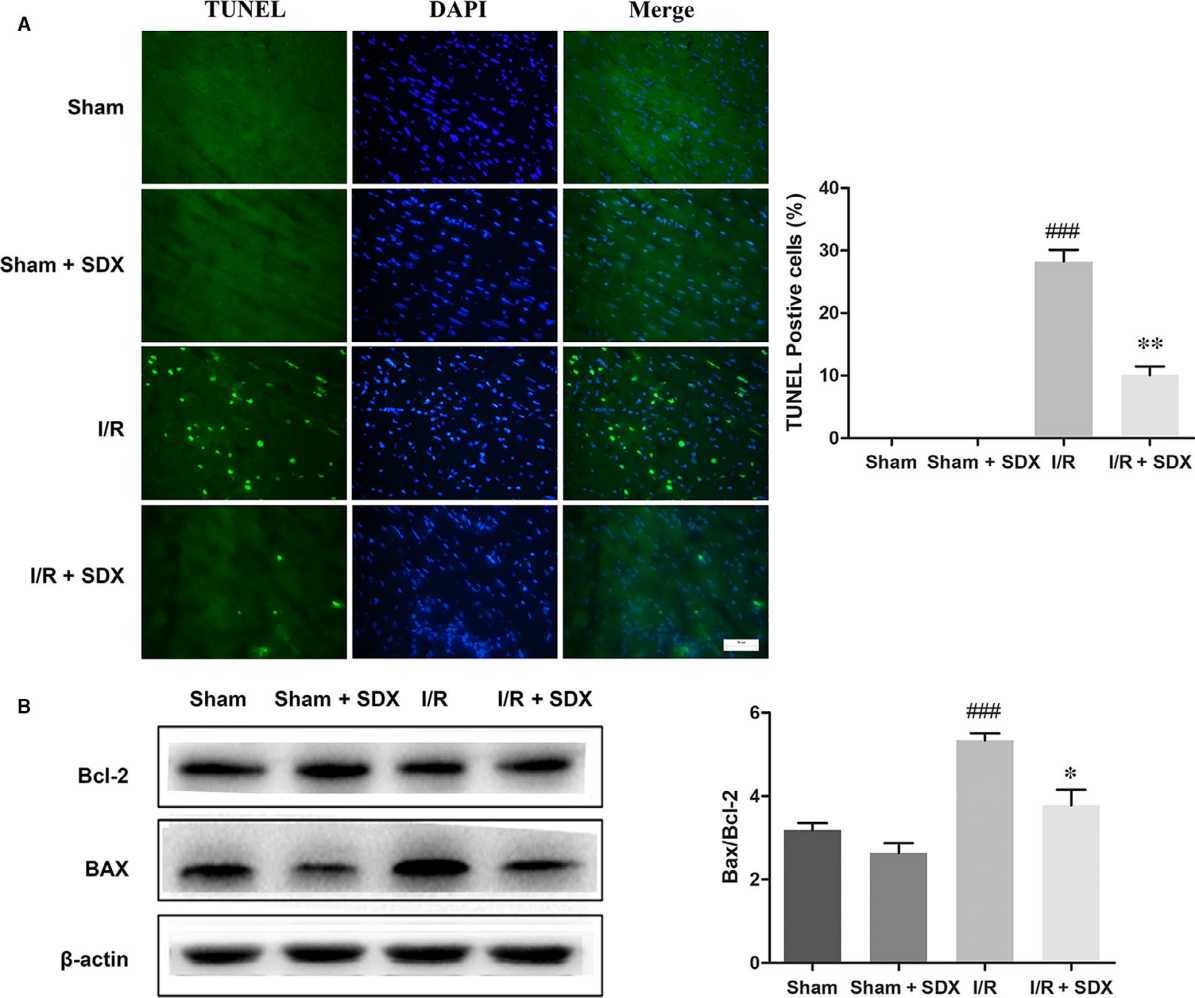
Sulodexide (SDX) reduces myocardial apoptosis and the BAX/Bcl‐2 proteins level in the hearts of mice after myocardial ischaemia/reperfusion. A, Representative images and quantitative analysis of terminal deoxynucleotidyl transferase‐mediated dUTP nick end labelling (TUNEL) immunofluorescence from the ischaemic area in the hearts of mice. B, The detection and optical density analysis of BAX/Bcl‐2 apoptosis proteins in the hearts. Data are shown as means ± SEM; ^###^ *P* < 0.001, I/R group vs Sham group, *P < 0.05, **P < 0.01, I/R + SDX group vs I/R group; n = 3 per group

Loss of cardiac cells in MI/R injury is caused by ER stress‐induced apoptosis.^30^ We investigated the effect of SDX on apoptosis during MI/R A TUNEL assay and showed that MI/R significantly increased apoptosis of cardiac cells, whereas SDX pre‐treatment ameliorated this effect (Figure 2A). The B‐cell lymphoma 2 (BCL2) protein family is associated with ER stress‐induced apoptosis^31^; within this family, Bcl‐2 plays an anti‐apoptotic and Bax a pro‐apoptotic role. As expected, MI/R increased Bax and decreased Bcl‐2 protein levels in cardiomyocytes and these effects were alleviated by SDX pre‐treatment (Figure 2B).

### 3.3 SDX exerted the cardioprotective effect by reducing ER stress and activating the PI3K/AKT pathway

**Figure 3:**
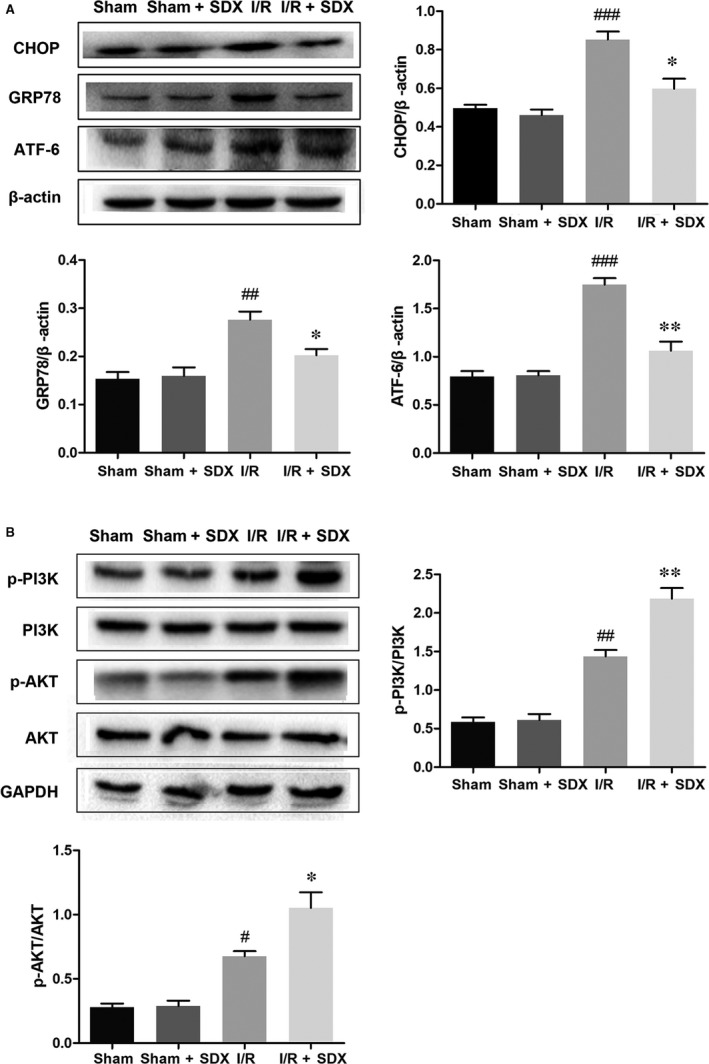
The effect of sulodexide (SDX) on endoplasmic reticulum stress‐related proteins in the hearts of mice after myocardial ischaemia/reperfusion. A, The protein expression levels and optical density analysis of C/EBP homologous protein (CHOP), glucose‐regulated protein 78 (GRP78) and activating transcription factor 6 (ATF‐6) in the hearts. B, The protein expression levels and optical density analysis of p‐PI3K, PI3K, p‐AKT and AKT in the hearts. Data are shown as means ± SEM; ^#^ *P* < 0.05, ^##^ *P* < 0.01, ^###^ *P* < 0.001, I/R group vs Sham group, *P < 0.05, **P < 0.01, I/R + SDX group vs I/R group; n = 3 per group

To assess the role of SDX in ER stress induced by MI/R, we assayed the expression of several ER stress‐related proteins and the activation state of the PI3K/Akt signalling pathway. Pro‐apoptotic transcription factor C/EBP homologous protein (CHOP), ER‐targeted cytoprotective chaperones glucose‐regulated protein 78 (GRP78) and activating transcription factor 6 (ATF‐6) were up‐regulated in the heart of mice with MI/R (Figure 3A); however, this effect was reversed by SDX pre‐treatment. Importantly, administration of SDX to sham mice did not affect the levels of these proteins. The PI3K/Akt pathway is an important survival pathway in a variety of cell types and is involved in the regulation of ER stress.^32^, ^33^ We examined the activity of the PI3K/Akt pathway in the hearts of mice with MI/R As expected, MI/R activated the PI3K/Akt pathway and the magnitude of this activation was enhanced by SDX pre‐treatment, as determined by Western blotting (Figure 3B).

### 3.4 SDX reduced oxidative stress‐induced apoptosis in H9C2 cells

**Figure 4:**
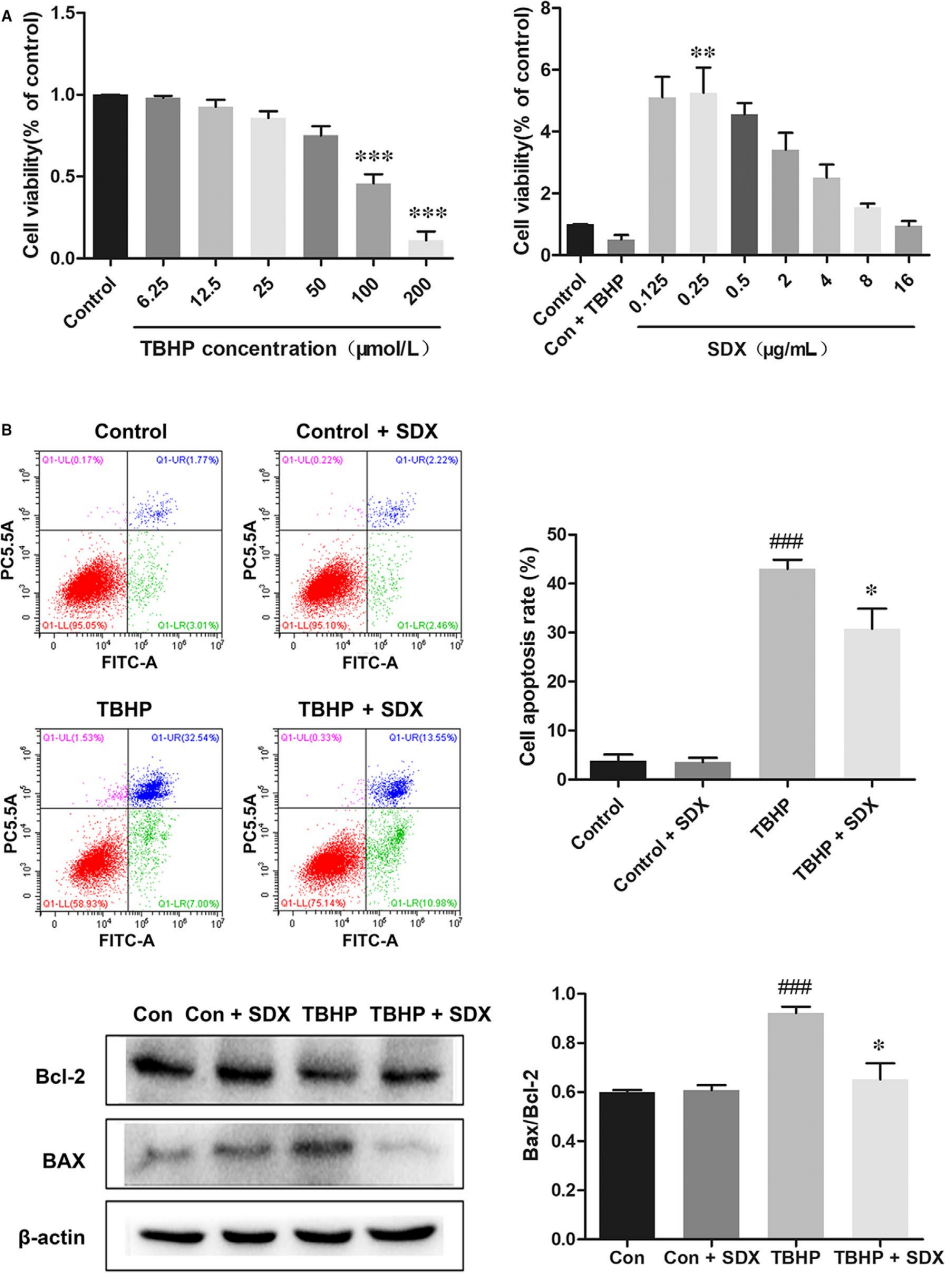
Sulodexide (SDX) inhibits apoptosis induced by tert‐butyl hydroperoxide (TBHP) in H9C2 cells. A, H9C2 cells were treated with different concentrations of TBHP for 12 h and then cell viability was assessed by the MTS assay. H9C2 cells were pre‐treated with different concentrations of SDX for 30 min, 100 µmol/L TBHP was added for an additional 12 h and then cell viability was assessed by the MTS assay. **P < 0.01, ***P < 0.001, different groups vs control group. B, H9C2 cells were pre‐treated with 0.25 µg/mL SDX for 30 min and then 100 µmol/L TBHP was added for an additional 12 h. Cells were then stained with Annexin V‐fluorescein isothiocyanate (FITC)/propidium iodide and detected by flow cytometry. The apoptosis ratio was quantified by BD FACS software. C, The detection and optical density analysis of BAX/Bcl‐2 apoptosis proteins in the H9C2 cells. Data are shown as means ± SEM; ^###^ *P* < 0.001, TBHP group vs control group; *P < 0.05, **P < 0.01, TBHP + SDX group vs TBHP group; n = 3 per group

We next evaluated the cardioprotective effect of SDX in vitro. Tert‐butyl hydroperoxide induces oxidative stress and apoptosis in H9C2 cells.^34^ Dose‐response experiments showed that the optimal concentrations of TBHP and SDX in H9C2 cells were 100 µmol/L and 0.25 µg/mL respectively (Figure 4A). Flow cytometry indicated that TBHP induced the apoptosis of H9C2 cells and this effect was reduced by SDX pre‐treatment (Figure 4B). Moreover, the Bax protein level was significantly increased by TBHP treatment but reduced by SDX pre‐treatment. In contrast, the Bcl‐2 level was decreased by TBHP treatment and increased by SDX pre‐treatment (Figure 4C).

### 3.5 SDX attenuated ER stress and activated the PI3K/AKT pathway in H9C2 cells

**Figure 5:**
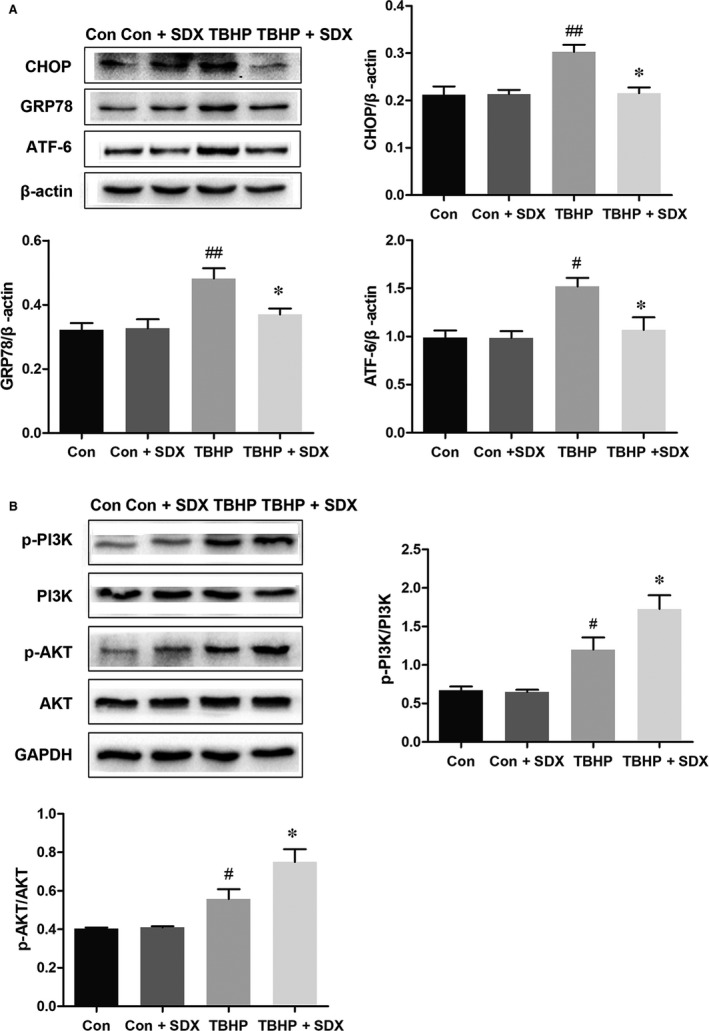
Sulodexide (SDX) attenuates endoplasmic reticulum stress‐related proteins induced by tert‐butyl hydroperoxide (TBHP) in H9C2 cells. A, The protein expression levels and optical density analysis of C/EBP homologous protein (CHOP), glucose‐regulated protein 78 (GRP78) and activating transcription factor 6 (ATF‐6) in H9C2 cells. B, The protein expression levels and optical density analysis of p‐PI3K, PI3K, p‐AKT and AKT in H9C2 cells. Data are shown as means ± SEM; ^#^ *P* < 0.05, ^##^ *P* < 0.01, TBHP group vs control group; *P < 0.05, TBHP + SDX group vs TBHP group; n = 3 per group

Based on the above results, we hypothesized that ER stress and the PI3K/Akt pathway are involved in the SDX‐mediated inhibition of apoptosis of H9C2 cells. Compared with the control group, the CHOP, GRP78 and ATF‐6 levels were significantly increased in the TBHP group and decreased in the SDX pre‐treatment group (Figure 5A). In addition, compared with the control group, the phosphorylated p‐PI3K and p‐Akt levels were increased in the TBHP‐treated group and the magnitudes of these increases were enhanced by SDX pre‐treatment (Figure 5B).

### 3.6 Inhibition of the PI3K/AKT pathway reversed the protective effect of SDX

**Figure 6:**
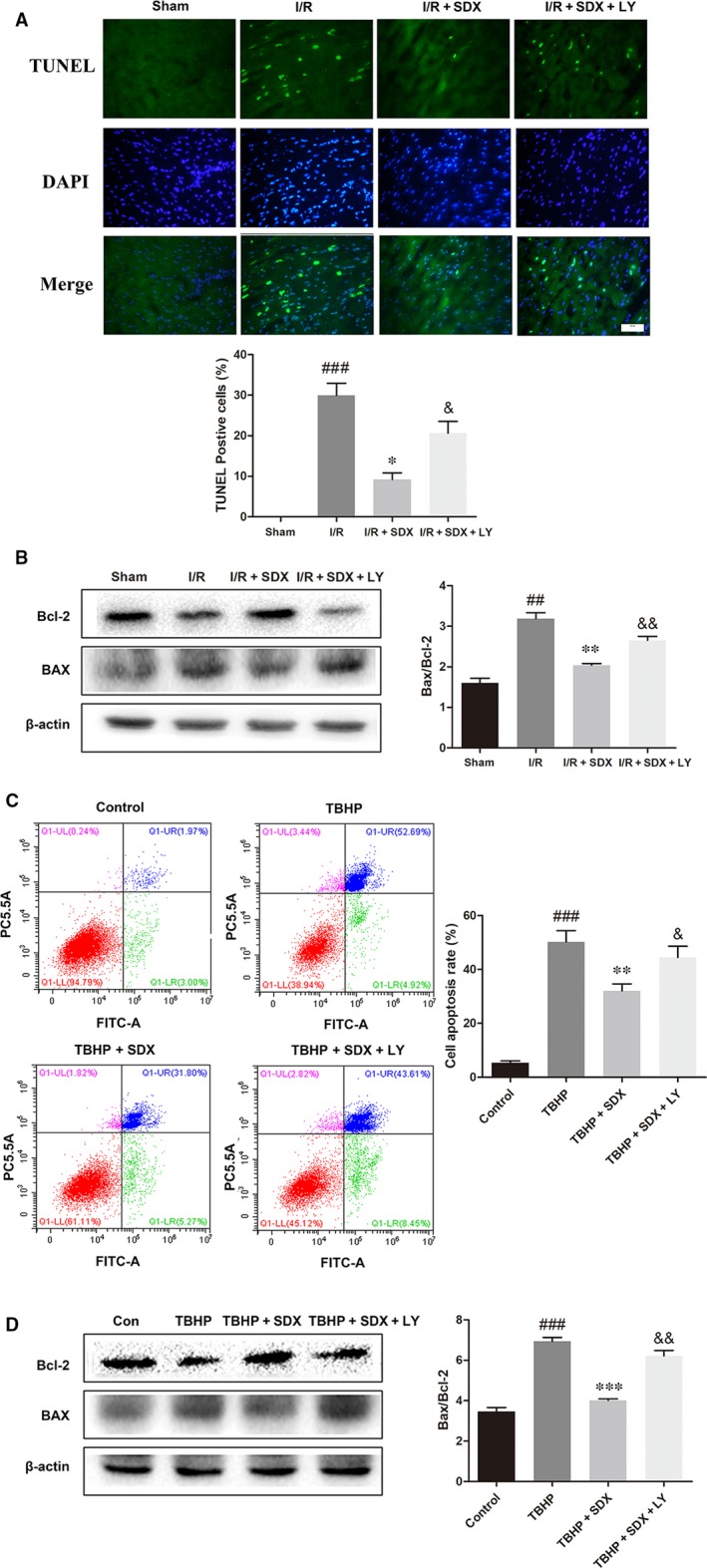
Inhibition of the PI3K/Akt pathway partially attenuates sulodexide (SDX)‐mediated reduction of myocardial cell apoptosis in vivo and in vitro. A, Representative images and quantitative analysis of terminal deoxynucleotidyl transferase‐mediated dUTP nick end labelling (TUNEL) immunofluorescence from the ischaemic area in the hearts of mice. B, The detection and optical density analysis of BAX/Bcl‐2 apoptosis proteins in the hearts. C, H9C2 cells were pre‐treated with 0.25 µg/mL SDX with or without the specific inhibitors LY294002 (20 µmol/L) for 30 min and then 100 µmol/L tert‐butyl hydroperoxide (TBHP) was added for an additional 12 h. Cells were collected and stained with Annexin V‐FITC/propidium iodide and detected by flow cytometry. The apoptosis ratio was quantified by BD FACS software. D, The detection and optical density analysis of BAX/Bcl‐2 apoptosis proteins in the H9C2 cells. Data are shown as means ± SEM; ^##^ *P* < 0.01, ^###^ *P* < 0.001, I/R group vs Sham group, TBHP group vs control group; *P < 0.05, **P < 0.01, ***P < 0.001, I/R + SDX group vs I/R group, TBHP + SDX group vs TBHP group; ^&^ *P* < 0.05, ^&&^ *P* < 0.01, I/R + SDX + LY294002 (LY) group vs I/R + SDX group, TBHP + SDX + LY vs TBHP + SDX; n = 3 per group

**Figure 7:**
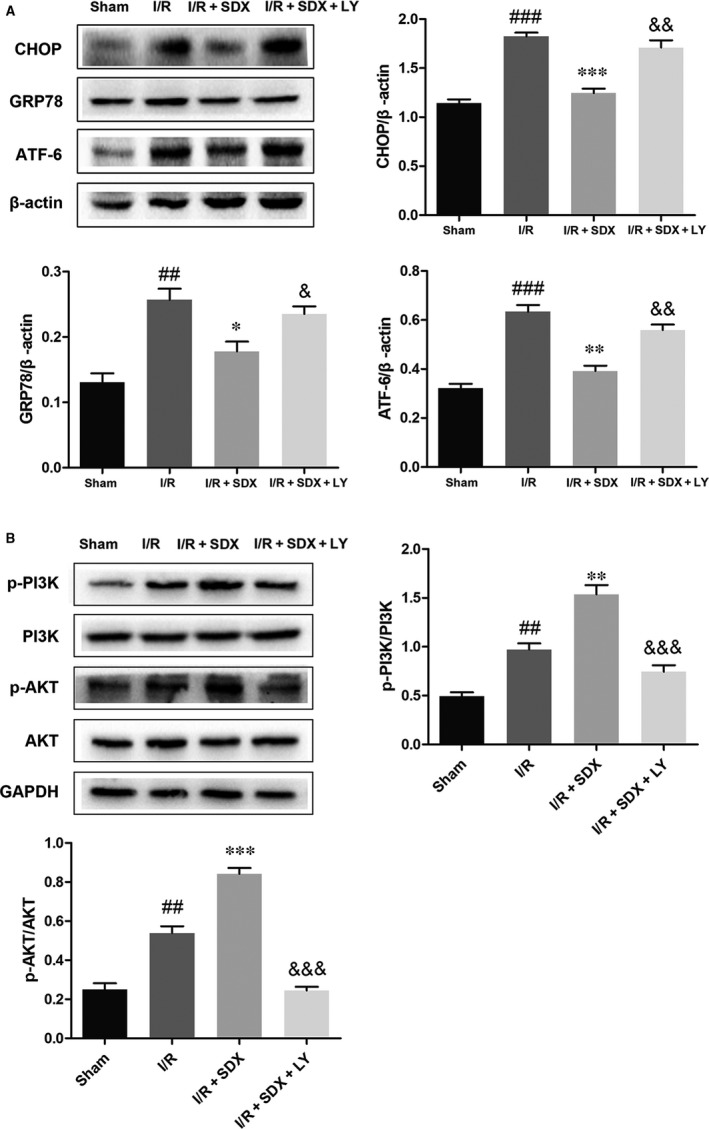
Inhibition of the PI3K/Akt pathway partially attenuates sulodexide (SDX)‐mediated reduction in the endoplasmic reticulum stress in mice. A, The protein expression levels and optical density analysis of C/EBP homologous protein (CHOP), glucose‐regulated protein 78 (GRP78) and activating transcription factor 6 (ATF‐6) in the hearts. B, The protein expression levels and optical density analysis of p‐PI3K, PI3K, p‐AKT and AKT in the hearts. Data are shown as means ± SEM; ^##^ *P* < 0.01, ^###^ *P* < 0.001, I/R group vs Sham group; *P < 0.05, **P < 0.01, ***P < 0.001, I/R + SDX group vs I/R group; ^&^ *P* < 0.05, ^&&^ *P* < 0.01, ^&&&^ *P* < 0.001, I/R + SDX + LY294002 (LY) group vs I/R + SDX group; n = 3 per group

**Figure 8:**
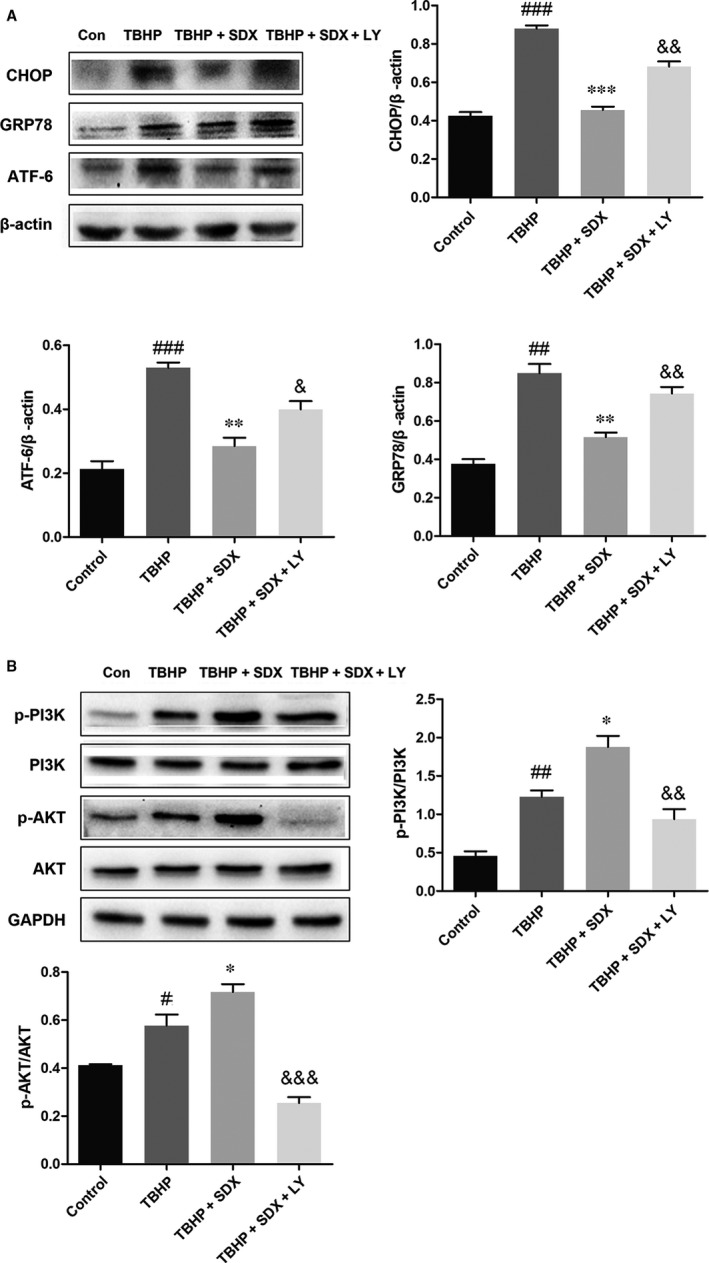
Inhibition of the PI3K/Akt pathway partially attenuates sulodexide (SDX)‐mediated reduction in the endoplasmic reticulum stress in H9C2 cells. A, The protein expression levels and optical density analysis of C/EBP homologous protein (CHOP), glucose‐regulated protein 78 (GRP78) and activating transcription factor 6 (ATF‐6) in H9C2 cells. B, The protein expression levels and optical density analysis of p‐PI3K, PI3K, p‐AKT and AKT in H9C2 cells. Data are shown as means ± SEM; ^#^ *P* < 0.05, ^##^ *P* < 0.01, ^###^ *P* < 0.001, tert‐butyl hydroperoxide (TBHP) group vs control group; *P < 0.05, **P < 0.01, ***P < 0.001, TBHP + SDX group vs TBHP group; ^&^ *P* < 0.05, ^&&^ *P* < 0.01, ^&&&^ *P* < 0.001, TBHP + SDX + LY294002 (LY) vs TBHP + SDX, n = 3 per group

To determine whether the SDX‐mediated suppression of ER stress‐induced apoptosis was related to the activation of the PI3K/Akt pathway, the PI3K inhibitor LY294002 was used. LY294002 significantly increased apoptosis compared with the MI/R + SDX group (Figure 6A). Moreover, LY294002 increased the Bax protein level and decreased that of Bcl‐2 compared with the SDX group in mice with MI/R injury (Figure 6B). Compared with the SDX pre‐treatment group, the levels of CHOP, GRP78 and ATF‐6 were increased, whereas those of p‐PI3K and p‐Akt were decreased, by LY294002 treatment (Figure 7A,B). Furthermore, LY294002 reversed the protective effect of SDX in H9C2 cells. Compared with the SDX pre‐treatment group, LY294002 significantly increased the apoptosis of H9C2 cells (Figure 6C). Similarly, the Bax protein level was increased and that of Bcl‐2 decreased in the LY294002 group compared with the control group (Figure 6D). Moreover, the LY294002‐induced changes in the CHOP, GRP78, ATF‐6 and p‐Akt protein levels in H9C2 cells were consistent with those in mice with MI/R (Figure 8A,B).

## 4 DISCUSSION

Myocardial ischaemia/reperfusion is the second most prevalent cardiovascular disease after acute myocardial infarction and is a leading cause of morbidity and mortality worldwide.^35^ Myocardial ischaemia/reperfusion is a long‐term myocardial injury secondary to inflammation, oxidative stress and apoptosis. Importantly, the death of cardiac cells caused by ER stress interferes with recovery from secondary damage.^36^ In this study, pre‐treatment with SDX protected cardiomyocytes from damage and reduced apoptosis in a mouse model of MI/R and in TBHP‐induced H9C2 cells. In addition, SDX ameliorated MI/R‐induced myocardial injury by reducing ER stress in a manner dependent on activation of the PI3K/Akt pathway.

Sulodexide exerts a protective effect on cardiovascular diseases by down‐regulating pro‐inflammatory cytokines and reducing deposition of C‐reactive protein.^16^, ^26^ However, whether SDX protects against MI/R injury was unknown. We report here that SDX decreased the level of the apoptosis‐related protein Bax and increased that of Bcl‐2 in TBHP‐treated H9C2 cells. In vivo, oral administration of SDX increased the SOD activity and decreased the MDA level, infarct size and apoptosis of cardiomyocytes in a mouse model of MI/R Therefore, SDX may protect against MI/R injury by promoting the survival of cardiomyocytes.

Endoplasmic reticulum stress leads to accumulation of unfolded or misfolded proteins. Moderate ER stress is sensed by transmembrane proteins and initiates an unfolded protein response.^37^ However, persistent or excessive ER stress up‐regulates the molecular chaperone GRP78 and activates apoptosis. CHOP is involved in ER stress‐induced apoptosis.^38^, ^39^ In this study, SDX reduced the expression of GRP78 after MI/R and decreased ATF‐6 and CHOP levels in vivo and in vitro. Thus, the effect of SDX on MI/R is mediated by down‐regulation of proteins related to the response to ER stress.

Phosphatidylinositol 3‐kinase/Akt signalling, a key component of the reperfusion injury salvage kinase (RISK) pathway, is involved in cardioprotection.^40^ Activation of the PI3K/Akt pathway attenuates sustained or severe ER stress‐induced cell death and plays a key role in cell survival.^41^, ^42^ In our study, the PI3K/Akt pathway was activated by MI/R. The magnitude of the activation was enhanced and myocardial damage and apoptosis were reduced, by SDX pre‐treatment. The PI3K inhibitor LY294002 partly alleviated the protective effect of SDX on cardiomyocytes. In addition, SDX down‐regulated the levels of ER stress‐related proteins and this effect was attenuated in part by LY294002. Therefore, SDX protects against MI/R‐induced cardiac damage via the PI3K/Akt pathway.

## 5 CONCLUSION

In conclusion, MI/R can induce myocardial apoptosis through ER stress and SDX pre‐treatment can significantly protect cardiomyocytes by inhibiting the expression of ER stress‐related proteins through the PI3K/Akt signalling pathway. In the present study, it was shown that treatment with SDX may be suitable for myocardial cell recovery from secondary injury.

## CONFLICTS OF INTEREST

The authors confirm that the content of this article has no conflicts of interest.

## AUTHOR CONTRIBUTION

All authors' contributions are in the order of ranking, Maoping Chu and Yuanhai Zhang are co‐corresponding authors.

## DATA AVAILABILITY STATEMENT

The data that support the findings of this study are available on request from the corresponding author. The data are not publicly available due to privacy or ethical restrictions.
